# Understanding of leukemic stem cells and their clinical implications

**DOI:** 10.1186/s12943-016-0574-7

**Published:** 2017-01-30

**Authors:** Xuefei Wang, Shile Huang, Ji-Long Chen

**Affiliations:** 1 CAS Key Laboratory of Pathogenic Microbiology and Immunology, Institute of Microbiology, Chinese Academy of Sciences, Beijing, 100101 China; 2 University of Chinese Academy of Sciences, Beijing, China; 3 Department of Biochemistry and Molecular Biology, Louisiana State University Health Sciences Center, Shreveport, LA USA; 4 College of Animal Sciences, Fujian Agriculture and Forestry University, Fuzhou, China

**Keywords:** Cancer stem cell, Leukemia, Leukemic stem cell, Surface markers, BM niche, ncRNAs, Clinical implications

## Abstract

Since leukemic stem cells (LSCs) or cancer stem cells (CSCs) were found in acute myeloid leukemia (AML) in 1997, extensive studies have been contributed to identification and characterization of such cell populations in various tissues. LSCs are now generally recognized as a heterogeneous cell population that possesses the capacities of self-renewal, proliferation and differentiation. It has been shown that LSCs are regulated by critical surface antigens, microenvironment, intrinsic signaling pathways, and novel molecules such as some ncRNAs. To date, significant progress has been made in understanding of LSCs, leading to the development of numerous LSCs-targeted therapies. Moreover, various novel therapeutic agents targeting LSCs are undergoing clinical trials. Here, we review current knowledge of LSCs, and discuss the potential therapies and their challenges that are being tested in clinical trials for evaluation of their effects on leukemias.

## Background

The existence of CSCs was firstly evidenced in AML [1], and has been now extended to a broad spectrum of solid tumors [2–8]. In 1994, Dick and colleagues [1] showed that only the leukemic cells expressing the same markers as normal adult hematopoietic stem cells (CD34^+^CD38^−^) could initiate hematopoietic malignancy, and termed these cells as leukemia-initiating cells, leukemic stem cells (LSCs), or cancer stem cells (CSCs) [1, 9, 10]. Importantly, such cell population possesses the capacities for self-renewal, proliferation and differentiation. Increasing evidence has demonstrated that LSCs are clinically relevant, indicating that therapies targeting LSCs in AML would improve survival outcomes [11].

Conventional anticancer strategy is a combination of surgery, chemotherapy and radiotherapy with allogeneic stem cell transplantation for eligible candidates [12–14]. However, elderly patients cannot tolerate such intense regimens, and patients usually face the risk of recurrence, metastasis and drug resistance. It is thought that these therapies predominantly target at a bulk tumor populations but leave CSCs behind. Importantly, these CSCs, with highly expressed ATP-binding cassette (ABC) transporters, have been shown to protect themselves from the attacks from chemotherapeutic agents [15–17]. Hence, the inefficient therapy of cancers is mainly attributed to the failure of elimination of the malignant CSCs. It is well recognized that development of CSC-selective therapies is important for treating CSCs-containing cancers [18]. In this review, we discuss the current understanding of LSCs. Also, we summarize various therapeutic agents targeting LSCs that are being studied in clinical trials.

## Genetic and epigenetic heterogeneities of LSCs

Leukemias are now viewed as aberrant hematopoietic processes initiated by rare LSCs, which arise from the transformation of hematopoietic stem cells (HSCs) or committed progenitor cells [19]. During the course of malignant transformation, LSCs acquire the capacity of self-renewal, proliferation and differentiation through continuous genetic and epigenetic alteration and clonal diversification. Thus, understanding how genetic and epigenetic heterogeneities develop in different leukemias has become an important area for cancer research. Although CSCs have been found in both leukemia and solid tumors, not all of CSCs in the solid tumors follow the heterogeneity model of LSC.

Increasing investigations using deep genome sequencing have identified many recurrent mutated genes critically implicated in the pathogenesis of human AML [20–27]. In 2013, the Cancer Genome Atlas Research Network analyzed the genome of 200 AML patients, and thoroughly defined the recurrent mutations in AML [28]. About 30 genes were identified to be mutated in more than 2% of patients. Remarkably, many of these mutated genes encode proteins that normally function at the epigenetic level, including modifications of DNA cytosine residues and post-translational modifications of histones. In addition, other studies have shown that clonal composition of AMLs appears to be changed quite markedly at both the genetic and epigenetic levels after therapy in relapsed disease [29–31].

Interestingly, it has been found that there is a sequential order for the acquisition of these mutations during leukemogenesis. For example, some researchers observed that somatic mutations in epigenetic modifiers that regulate cytosine methylation, such as *DNMT3A* (DNA methyltransferase 3 alpha), *IDH1/2* (isocitrate dehydrogenase 1/2) and *TET2* (tet methylcytosine dioxygenase 2), occur early in pre-leukemic HSCs [32–34]. However, other somatic mutations in signaling pathways that drive proliferation, such as *NPM1* (nucleophosmin 1), *FLT3-ITD* (internal tandem duplication of the gene *FLT3*) and *KRAS/NRAS* (Kirsten rat sarcoma viral oncogene homolog/neuroblastoma rat sarcoma viral oncogene homolog), are later events in AML transformation [35]. These results suggest that disruption of epigenetic patterning is likely an early and prominent event during leukemogenesis.

In order to characterize the expression profile of LSCs in chronic myeloid leukemia (CML), Gerber and colleagues performed genome-wide transcriptome analysis of CML LSCs using exon microarrays [36]. They identified 97 genes that are differentially expressed between CML LSCs and normal HSCs. Further analysis revealed dysregulation of proliferation, differentiation and signaling pathways in CML LSCs. These data may provide potential therapeutic targets unique to CML LSCs.

## Surface molecules and microenvironment of LSCs and their clinical implications

### Cell surface molecules of LSCs

The AML LSCs were the first reported and best characterized type of CSCs, and they specifically display CD34^+^CD38^−^ cell surface markers [1, 9, 10]. However, subsequent studies showed that the surface markers of AML LSCs are considerably heterogeneous [37–47]. For example, AML LSCs were found not only in Lin^−^/CD38^−^ fractions but also in CD34^−^, Lin^+^, CD38^+^, and CD45RA^+^ fractions [45]. It was also found that true AML LSCs in the CD34^+^/CD38^−^ fractions, originally described by Bonnet and Dick, were very rare and comprised a hierarchy of cells with different self-renewal potential [46]. In addition, some surface markers of AML LSCs (CD34^+^, CD38^−^, CD71^−^, and HLA-DR^−^) are shared with normal HSCs, and others (Lin^+^, CD38^+^, CD45RA^+^) are associated with normal committed progenitors [38, 45]. These findings stirred up a debate about whether AML LSCs are derived from the normal HSCs or from the committed progenitor cells. On the other hand, the surface markers of LSCs are heterogeneous, which makes hard for classification of LSCs and even LSCs-targeted treatment in clinics.

Recently, great progress has been made in understanding of LSC surface markers and their clinical applications, especially in AML cases. Firstly, a number of critical surface markers unique to AML LSCs have been identified. For example, it has been revealed that CD90 and CD117 are deficient in AML LSCs [39], while CD123 [42, 48], TIM3 [44, 49], CD47 [50, 51], CD96 [52], CLL-1 [53, 54], and IL-1 receptor accessory protein (IL1RAP) [55] are highly expressed in AML LSCs. Targeting these surface markers is a promising strategy for eradicating AML LSCs. Previous studies have shown that CD123 (IL-3 receptor α chain) was preferentially expressed in the CD34^+^/CD38^−^ AML cells, as compared with normal HSC samples. Pretreatment of AML cells with anti-CD123 monoclonal antibody 7G3 resulted in decreased engraftment when they were injected into a xenograft model [42, 48]. To date, phase I clinical trials (NCT00401739 and NCT01632852) of using monoclonal antibody targeting CD123 (CSL360 and improved CSL362) [48] have been tested in CD123^+^ AML patients. Moreover, other monoclonal antibodies targeting CD47 [56, 57], CD96 [52, 58], TIM3 [44, 49] and CLL-1 [54, 59] have also been investigated in pre-clinical models for their ability to eliminate primary AML LSCs. It is worth mentioning that Gemtuzumab Ozogamicin, an anti-CD33 antibody, is the first monoclonal antibody approved by the Food and Drug Administration (FDA) of the USA in 2000 for the treatment of AML, although it may not specifically target LSCs [60].

Secondly, increasing novel therapies are continuously developed to specifically target these surface antigens of LSCs and are undergoing in clinical trials in AML cases. Besides monoclonal antibodies mentioned above [61, 62], these new therapies include both bi-specific and tri-specific antibody fragments [63, 64], immunotoxins [65], chimeric antigen receptor modified T-cells (CAR T-cells) [66], and nano-particles containing surface markers-targeted medication [67]. Notably, DT388IL3 (SL-401) is a recombinant immunotoxin that is created by fusing diphtheria toxin with a ligand targeting IL-3 receptor. At present, DT388IL3 (SL-401) undergoes phase I/II trials (NCT02113982 and NCT02270463) in AML [65] (Table 1).

**Table 1:** Anti-LSCs agents that are undergoing in AML clinical trials

Targets	Name of agents	Property of agents	Stage of clinical trials	Clinical trial identifier	References
Cell surface antigens					
CD123	CSL360	Monoclonal antibody	Phase I	NCT00401739	[42, 48]
	CSL362	Monoclonal antibody	Phase I	NCT01632852	[48]
	DT388IL-3 (SL-401)	Immunotoxin	Phase I/II	NCT02113982,NCT02270463	[65]
Signaling pathways					
PI3K	CAL-101 (Idelalisib)	Inhibitor of PI3K	Phase I	NCT00710528	[114]
AKT	Perifosine	Inhibitor of AKT	Phase I	NCT00301938	[184, 185]
	MK-2206	Inhibitor of AKT	Phase II	NCT01253447	[186, 187]
mTOR	Everolimus(RAD001)	Inhibitor of mTOR	Phase II	NCT00762632	[188]
	Temsirolimus	Inhibitor of mTOR	Phase II	NCT00775593	[189, 190]
NF-κB	Bortezomib	Inhibitor of IκB	Phase I/II	NCT00651781,NCT00742625	[191, 192]
Wnt	CWP232291	Inhibitor of β-catenin	Phase I	NCT01398462	/
Microenvironment					
CXCR4	Plerixafor(AMD3100)	Antagonist of CXCR4	Phase I/II	NCT00990054,NCT00822770	[75–77, 193]

### Microenvironment associated with LSCs

Under normal conditions, HSCs rely on the interactions with the bone marrow (BM) niche, which is critical for their proper function and maintenance [68]. The remodeling of the BM niche is commonly observed in blood malignancies. There is evidence that growth of leukemic cells disrupts the BM niches of normal hematopoietic progenitor cells and creates a microenvironment hospitable for them [69]. Within such microenvironment, LSCs are able to communicate with BM stromal cells through cytokines, chemokines and intracellular signals initiated by cellular adhesion [70, 71]. Importantly, these signals influence the ability of LSCs to self-renew, maintain their quiescence, and prevent apoptosis. In addition, the BM niche provides two distinct microenvironmental zones (the osteoblastic niche and vascular niche) that likely regulate the cycling of LSCs [71–73]. Thus, blocking the interactions between LSCs and their microenvironment represents a promising strategy to disrupt LSC homeostasis and restore normal hematopoiesis.

One of such strategies is to dislodge LSCs from their protective BM niche and thus sensitize the LSCs to conventional chemotherapies. It has been demonstrated that LSCs migrate into and remain within the BM niche through the interaction between C-X-C chemokine receptor type 4 (CXCR4) and stromal cell derived factor-1 (SDF-1α), also known as C-X-C motif chemokine 12 (CXCL12) [74]. Recently, manipulating the CXCL12-CXCR4 axis using Plerixafor (AMD3100) in relapsed AML has been reported as a safe strategy in phase I/II clinical trials (NCT00990054 and NCT00822770) [75–78]. Additionally, ligation of the adhesion molecules CD44 [79] and vascular cell adhesion molecule 1 (VCAM-1) [80] with their monoclonal antibodies has already been tested in the clinic. Other strategies like altering BM niche remodeling and inflammatory microenvironment, such as targeting pro-inflammatory cytokines tumor necrosis factor alpha (TNFα), IL-1, and IL-6, might be very promising but mainly at pre-clinical stages [81].

## Intracellular molecules and signaling of LSCs

### Critical signaling pathways involved in regulation of LSCs

LSCs are characterized by limitless self-renewal, proliferation and differentiation. A set of critical genes impact these functional properties through a wide range of cellular pathways and processes, which have been described in detail by many groups [13, 19, 71, 82]. Signaling pathways such as Wnt/β-catenin [83–89] and Hedgehog [90–92] play important role in regulating self-renewal of LSCs. These signaling pathways are also critically required for the development of normal HSCs [93]. In addition, it is thought that LSCs can evade apoptosis by up-regulating NF-κB (nuclear factor kappa-light-chain-enhancer of activated B cells) [94, 95] or by down-regulating Fas/CD95 [96]. Here, we review some key signaling pathways involved in the regulation of survival and self-renewal of LSCs.

The well-known Wnt/β-catenin signaling pathway plays fundamental role in maintaining CSC populations. The activation of Wnt/β-catenin pathway leads to the translocation of β-catenin into the nucleus, where it induces the expression of target genes such as c-Myc, c-Jun and cyclin D1 [97–101]. Various experiments have demonstrated that Wnt/β-catenin signaling pathway acts as a key regulator in controlling proliferation, survival, and differentiation of hematopoietic cells [99, 102]. Aberrant activation of Wnt/β-catenin signaling pathway has also been found in both AML [87–89] and CML [83, 84]. Subsequent studies have shown that Wnt/β-catenin signaling pathway is required for efficient self-renewal of LSCs, indicating that it is an attractive therapeutic strategy to target Wnt/β-catenin signaling in AML and CML [84, 85]. In addition, it has been documented that Wnt signaling pathway and the polycomb-group protein BMI1 (B lymphoma Mo-MLV insertion region 1 homolog) are involved in the expansion of LSCs [103–105].

Janus kinase (JAK)/signal transducer and activator of transcription (STAT) and phosphatidylinositide 3-kinase (PI3K)/protein kinase B (AKT) are two crucial signaling pathways that have been implicated in LSC survival and multiple cancer formation. It is well established that malignant transformation of many cell types, especially hematopoietic cells, involves the dysregulation of JAK/STAT and/or PI3K/AKT that regulate cellular proliferation and survival. For example, there is considerable evidence showing that aberrations in these signaling pathways are associated with numerous leukemias. In CML, JAK/STAT/PIM (proviral insertion in murine) and PI3K/AKT/mTOR (mammalian/mechanistic target of rapamycin) pathways are constitutively activated by Bcr-Abl, a non-receptor tyrosine kinase, resulting in uncontrolled cellular proliferation [12, 106–108]. Bcr-Abl can also cause tyrosine phosphorylation of suppressors of cytokine signaling 1 and 3 (SOCS-1 and SOCS-3), two potent suppressors of JAK/STAT signaling, and thereby diminish their inhibitory effects on JAK/STAT activation [109]. Interestingly, PI3K mutation and AKT1 (E17K) mutation has been identified in a variety of tumors. AKT1 (E17K) mutant, a constitutively activated form of AKT1, can significantly promote tumorigenesis [110]. In addition, it was observed that other members of the PI3K/AKT/mTOR pathway, such as PTEN (phosphatase and tensin homolog) and mTOR, function in the maintenance of LSCs [111]. Recently, we have shown that there exists a crosstalk between JAK/STAT/PIM and PI3K/AKT/mTOR pathways that converge on eukaryotic translation initiation factor 4B (eIF4B) to regulate the survival of Abl transformants [112, 113].

In brief, increasing evidence has suggested that multiple signaling pathways are involved in the development of LSCs. Profound elucidation of the intricate pathway network in LSCs is significant in understanding of LSCs and designing precise treatment of leukemia through targeting LSCs. Currently, various clinical trials are in process to test the efficacy of agents targeting intracellular proteins and pathways associated with LSCs. For example, clinical studies of the drug CAL-101, an inhibitor of PI3K, showed remarkable success in chronic lymphocytic leukemia (CLL). It has also been found that CAL-101 has some effects on tumor microenvironment [114]. Additionally, other inhibitors targeting PI3K/AKT/mTOR, NF-κB and Wnt signaling in the clinic are listed in Table 1.

### Functional involvement of non-coding RNAs in malignant hematopoiesis

**Fig. 1:**
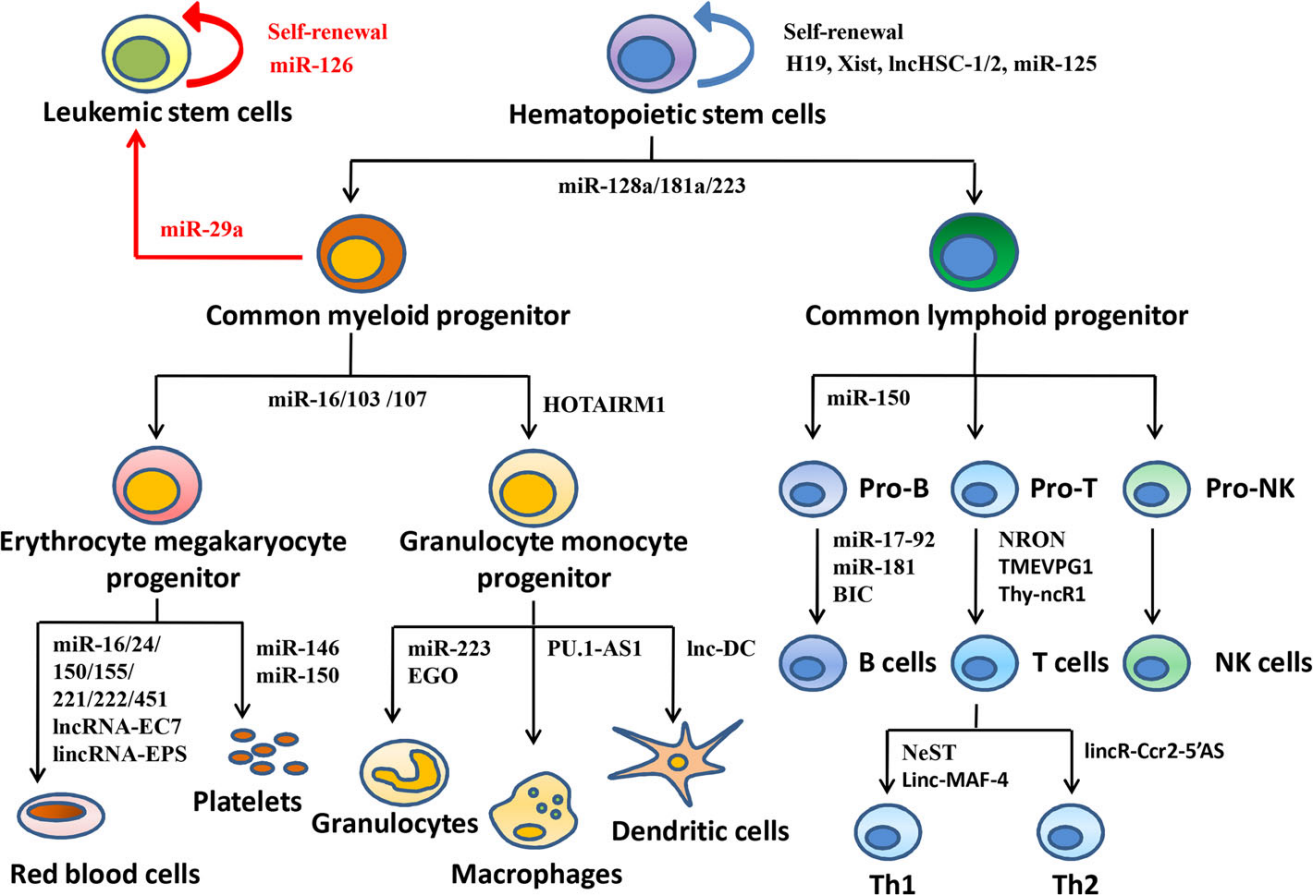
Involvement of miRNAs and lncRNAs in normal and malignant hematopoiesis. miRNAs and lncRNAs regulate almost every step of development and differentiation of hematopoietic cells during both normal and malignant hematopoiesis. Dysregulation of the ncRNAs (in red color) is associated with transformation of hematopoietic cells

Non-coding RNAs (ncRNAs), such as microRNAs (miRNAs) and long non-coding RNAs (lncRNAs), play critical roles in multiple biological processes [115–119]. Aberrant expression and functioning of these ncRNAs have been shown to be associated with various cancers and cancer stem cells [120–125]. Here, we highlight several miRNAs and lncRNAs as key regulators in hematopoietic cells and LSCs (Fig. 1).

#### Regulation of hematopoietic malignancies by miRNAs

miRNAs are 18–22 nucleotides ncRNAs that generally regulate gene expression by promoting mRNA degradation or inhibiting mRNA translation [126, 127]. During the tumorigenesis, some miRNAs act as oncogenes, whereas others function as tumor suppressors [128–132]. They can regulate cell growth, proliferation, survival, migration and invasion of cancer cells. Notably, the roles of well-known miRNAs in normal and malignant hematopoiesis have been extensively reviewed [133–137]. These miRNAs regulate almost every step of development and differentiation of both normal hematopoietic cells and LSCs.

MiR-125 is a highly conserved miRNA. There are three homologs of miR-125 (hsa-miR-125b-1, hsa-miR-125b-2 and hsa-miR-125a) in human [136]. Previous investigations have revealed that highly expressed miR-125 enhances self-renewal and survival of HSCs, and dysregulation of miR-125 occurs in multiple hematopoietic malignancies [138–142]. In particular, miR-125 is implicated in hematopoiesis through the p53-involved regulation network [143].

Recently, Lechman and colleagues have shown that miR-126 preserves AML LSC quiescence and promotes chemotherapy resistance by targeting the PI3K/AKT/mTOR signaling pathway [144]. Interestingly, reduction of miR-126 level impairs LSC maintenance, but it plays an opposing role in normal HSCs [144]. In addition, functional involvement of miR-29a has also been found in AML LSCs [134]. Previous experiments demonstrated that miR-29a was highly expressed in AML samples. Furthermore, results exhibited that miR-29a can promote proliferation of hematopoietic progenitor, and transform AML by converting myeloid progenitors into LSCs [134].

#### Involvement of lncRNAs in leukemogenesis

Over the past decade, increasing numbers of lncRNAs have been identified and recognized as novel regulators that are implicated in various cellular processes. LncRNAs are generally more than 200 nucleotides in length, and modulate gene expression through interaction with DNAs, RNAs and proteins [145–147]. They function at multiple levels, including gene transcription, post-transcriptional processing, RNA translation, and epigenetic modifications [148]. It has been reported that some lncRNAs are involved in the regulation of CSCs [149–152]. For example, the lncRNA, named lncTCF7, has been identified to promote liver CSC self-renewal and tumor propagation by activating Wnt signaling [149]. Moreover, many lncRNAs have been seen to be associated with normal hematopoietic cells and various types of leukemia [118, 153–160] (Fig. 1).

Dysregulation of lncRNA H19 has been observed in various tumors, including Bcr-Abl-induced leukemia [161–164]. H19 acts as dual regulators in different cancers (either as an oncogene or a tumor suppressor) and also serves as a precursor for miR-675, known to down-regulate the tumor suppressor gene *RB* in human colorectal cancer [165–167]. Importantly, H19 is highly expressed in long-term HSCs (LT-HSCs). H19-deficiency results in activation of the insulin-like growth factor 2 (IGF2)-IGF1 receptor pathway, leading to increased proliferation and decreased long-term self-renewal of HSCs [168].

Recently, Guo et al. have comprehensively analyzed the expression of lncRNAs in human CML cells [158]. Notably, a lncRNA termed lncRNA-BGL3 was highly induced in response to silence of Bcr-Abl expression or inhibition of Bcr-Abl kinase activity in K562 cells and leukemic cells derived from CML patients. Furthermore, lncRNA-BGL3 functions as a competitive endogenous RNA (ceRNA) to cross-regulate PTEN expression, thereby modulating leukemic cell survival. Thus, lncRNA-BGL3 has been identified as a tumor suppressor in Bcr-Abl-mediated cellular transformation.

To date, miRNAs and lncRNAs have been confirmed by increasing evidence as functional mediators in cancer cells and cancer stem cells. Some cancer-associated ncRNAs are currently considered as biomarkers for patient prognosis and potential therapeutic agents for particular cancers [128, 129, 169–181]. For example, MRX34, the first miRNA mimic, entered phase I clinical trials in patients with advanced hepatocellular carcinoma in 2013 [169]. In AML, Dorrance et al. have observed that miR-126 enriches in AML LSCs and contributes to the long-term maintenance and self-renewal of LSCs. Treatment with novel nano-particles containing antagomiR-126 results in reduction of LSCs in vivo [181]. Therefore, better understanding of the mechanisms underlying functional involvement of miRNAs and lncRNAs in LSC development and leukemogenesis is of great importance for precise treatment of hematopoietic malignancies.

## Conclusion

Over the past two decades, the function and phenotype of LSCs have been continuously defined. Furthermore, numerous studies provide accumulating evidence that there exist CSCs in a variety of solid tumors [182, 183]. Importantly, these progresses have led to the development of many novel therapeutic strategies targeting CSCs. Here, we have reviewed the current understanding of LSCs both in intrinsic and extrinsic aspects, and discussed the promising therapeutics that is being tested in clinical trials. Although identification and characterization of LSCs have renewed leukemia research and helped develop diverse clinical therapeutic strategies, some tough challenges for LSCs-based leukemia therapy still remain. One of the greatest challenges is early and efficient identification of LSCs in diverse leukemia patients. Moreover, better understanding of LSCs development and differentiation is critically required for clinical implications of the strategies targeting such cell populations. Precise mechanisms by which extracellular and intracellular molecules and their signaling regulate LSCs also remain to be determined. Therefore, further efforts are needed to identify more specific biomarkers of LSCs, determine specific targets and thereby develop efficient LSCs-based treatment of leukemia.

## Abbreviations

ABC transporters: ATP-binding cassette transporters
AML: Acute myeloid leukemia
BM niche: Bone marrow niche
BMI1: B lymphoma Mo-MLV insertion region 1 homolog
CAR T-cells: Chimeric antigen receptor modified T-cells
CEBPE: CCAAT/enhancer binding protein epsilon
CEPBA: CCAAT/enhancer binding protein alpha
CeRNA: Competitive endogenous RNA
CLL: Chronic lymphocytic leukemia
CML: Chronic myeloid leukemia
CSCs: Cancer stem cells
CXCL12: C-X-C motif chemokine 12
CXCR4: C-X-C chemokine receptor type 4
*DNMT3A*: DNA methyltransferase 3 alpha
eIF4B: Eukaryotic translation initiation factor 4B
FDA: Food and Drug Administration
*FLT3-ITD*: Internal tandem duplication of the gene FLT3
HSCs: Hematopoietic stem cells
*IDH1/2*: Isocitrate dehydrogenase 1/2
IGF2: Insulin-like growth factor 2
IL1RAP: IL-1 receptor accessory protein
JAK/STAT: Janus kinase/signal transducer and activator of transcription
KRAS/NRAS: Kirsten rat sarcoma viral oncogene homolog/neuroblastoma rat sarcoma viral oncogene homolog
lncRNAs: Long non-coding RNAs
LSCs: Leukemic stem cells
LT-HSCs: Long-term HSCs
miRNAs: microRNAs
mTOR: mammalian/mechanistic target of rapamycin
ncRNAs: non-coding RNAs
NF-κB: Nuclear factor kappa-light-chain-enhancer of activated B cells
NPM1: Nucleophosmin 1
PI3K/AKT: Phosphatidylinositide 3-kinase/protein kinase B
PIM: Proviral insertion in murine
PTEN: Phosphatase and tensin homolog
SDF-1α: Stromal cell derived factor-1
SOCS-1 and SOCS-3: suppressors of cytokine signaling 1 and 3
TET2: Tet methylcytosine dioxygenase 2
TNFα: Tumor necrosis factor alpha
VCAM-1: Vascular cell adhesion molecule 1
